# Granulomatous mastitis, erythema nodosum, and polyarthritis: a case report

**DOI:** 10.1186/s13256-022-03327-5

**Published:** 2022-04-05

**Authors:** Leanna Laor, Suhas Ganguli, Esra Fakioglu

**Affiliations:** 1 Flushing Hospital Medical Center, 4500 Parsons Boulevard, Flushing, NY 11355 USA; 2 Pediatric Rheumatology Marshfield Clinic, 1000 North Oak Ave, Suite 1A1,, Marshfield, WI 5449 USA

**Keywords:** Granulomatous mastitis, Erythema nodosum, Polyarthritis

## Abstract

**Background:**

Granulomatous mastitis is a rare inflammatory disease of the breast, typically seen in woman of child-bearing age. No definitive etiology has been described. In rare instances, this condition has been reported to be associated with extramammary manifestations such as erythema nodosum and arthritis. We describe this rare condition in an adolescent female.

**Case presentation:**

A 16-year-old, Hispanic female presented with right-sided painful breast swelling, polyarthritis, and erythema nodosum on bilateral shins and lower thighs. Physical examination was negative for lymphadenopathy and pulmonary, gastrointestinal, and cardiovascular findings. Ophthalmologic examination for uveitis and serologic tests for autoimmune diseases were negative. Diagnosis of idiopathic granulomatous mastitis was made by exclusion of other etiologies and conditions. Confirmation was made by histopathologic examination demonstrating noncaseating granuloma within breast lobules with neutrophils and microabscess formation. After wide local excision and a short course of trimethoprim–sulfamethoxazole, our patient was placed on naproxen and prednisone, the latter being tapered off over 3 months, with steady and complete resolution of all symptoms.

**Conclusion:**

This is the first reported case of idiopathic granulomatous mastitis in a pediatric patient who also had extramammary manifestations, including erythema nodosum and polyarthritis. In this case-based review, we summarize the phenotype, risk factors, prognosis, and treatment options of this rare condition, chiefly to make the readers cognizant of such a diagnostic possibility in similar clinical presentation in the future.

## Background

Granulomatous mastitis (GM) is a chronic, rare, inflammatory condition of the breast of either idiopathic or secondary etiology. The condition typically affects non-white (Middle Eastern or Hispanic) women, of reproductive age, with a mean age of 37 years [1, 2]. Although Veyssierie *et al*. first described the condition in 1967 [3], Wolloch and Kessler first established the condition in 1972 [4].

Granulomatous mastitis accounts for 1.6% of all biopsy specimens and, due to demographic and clinical overlap, can be misdiagnosed as breast carcinoma [5]. Histologic findings include noncaseating granulomas centered on lobules [1–5]. Various infectious etiologies (mycobacteria, parasitic, mycotic, viral), autoimmune and connective tissue diseases (granulomatosis with polyangiitis, sarcoidosis, rheumatoid arthritis), trauma, diabetes, hyperprolactinemia, and alpha-1 antitrypsin deficiency, among other conditions, have been implicated in secondary cases of granulomatous mastitis [6].

Granulomatous mastitis is reported to be associated with polyarthritis and/or erythema nodosum as extramammary manifestations [6]. This particular presentation creates the need to exclude differential diagnoses such as sarcoidosis, granulomatosis with polyangiitis, and inflammatory bowel disease.

Studies on this condition, focusing on its etiology and particularly on its treatment, have been centered on the adult population, where the limited number of cases precludes a uniform consensus on management. We report on the first such case in the pediatric age group.

## Case presentation

A 16-year old, US-born previously healthy, fully immunized, Hispanic female presented to the emergency department with complaints of swelling of the right breast for 4 weeks duration. In addition to breast findings, she reported pain and swelling of the left wrist, left elbow, bilateral knees, and ankles in an additive pattern for 4 weeks. She was prescribed multiple courses of antibiotics, including amoxicillin, clindamycin, and trimethoprim–sulfamethoxazole, with no relief of symptoms. Two days after starting clindamycin, the patient developed painful, palpable red lesions on the shins and lower thighs bilaterally. She reported no history of trauma, fever, weight loss, night sweats, cough, shortness of breath, irregular bowel patterns, or changes in stool pattern. She denied recent vaccination, travel, exposure to animals, and consumption of exotic foods.

**Fig. 1:**
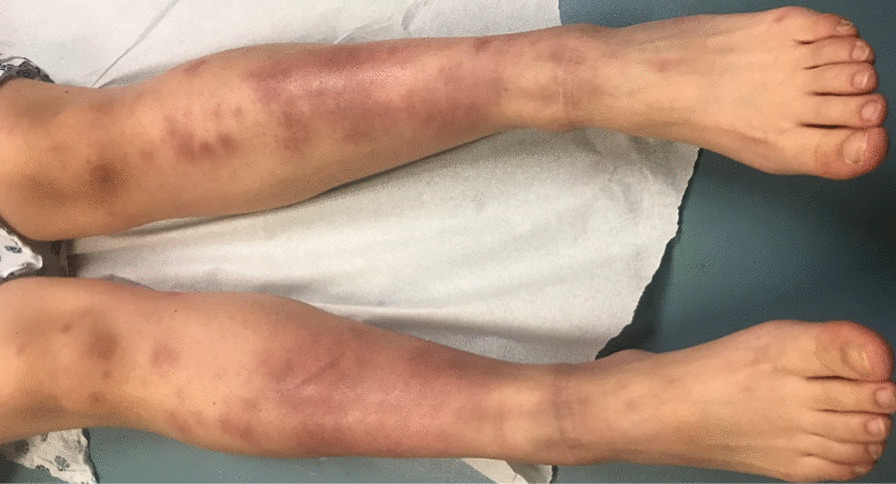
Erythema nodosum present on lower thighs and shins bilaterally in various stages of evolution

Upon admission, the right breast was noted to have a firm, poorly circumscribed area of induration located at the upper and lower outer quadrant, measuring approximately 11 cm in diameter, with an approximately 3 cm erythematous, tender patch with central fluctuation located above the areola. There were no skin changes or abnormalities of the nipple–areola complex, such as erythema, dimpling, peau-de-orange, nipple inversion, or drainage. The left breast was noted to be normal. Additionally, she had active arthritis of the bilateral knees and ankles. Examination of her lower extremities showed erythema nodosum on bilateral shins and lower third of thighs at different stages of evolution and peeling of overlying skin (Fig. 1).

She was continued on trimethoprim–sulfamethoxazole, prescribed prior to admission by another provider with suspicion of breast abscess, and placed on naproxen for the arthritis. Results of laboratory tests performed during admission can be found in Tables 1–3.

**Table 1:** Hematologic workup performed on the patient

Hemoglobin (g/dL)	11 (11.5–16)
Hematocrit (%)	34.3 (35–47)
WBC (×10^3^/μL)	10.6 (3.8–11)
Polymorphonuclear (%)	77 (40–70)
Lymphocytes (%)	13 (22–62)
Monocytes (%)	9 (0–6)
Eosinophils (%)	1 (0–5)
Platelets (×10^3^/μL)	291 (130–400)
ESR (mm per hour)	37 (0–25)

Pertinent findings included an elevated ESR. *WBC*: White Blood Cell; *ESR*: Erythrocyte Sedimentation Rate

**Table 2:** Infectious disease as well as endocrine/metabolic workup

Anti-HIV I, II	Negative
TB QuantiFERON	Negative
Blood culture	Negative
Culture of body fluid (breast collection)	Negative
TSH (μU/mL)	1.410 (0.47–4.68)
Vit D 25 (OH) (ng/mL)	27.5 (30–100)
Vit D 1,25 (OH) (pg/mL)	50 (19–83)

*Anti-HIV I, II*: HIV Antibody I, II; *TB Quantiferon*: Tuberculosis Quantiferon; *TSH*: Thyroid Stimulating Hormone

**Table 3:** Rheumatologic workup

ANA	Negative
p-ANCA (MPO)	Negative
c-ANCA (PR 3)	Negative
Smith antibody	< 1
Smith/RNP antibody	< 1
Ds DNA antibody	< 1
C3 complement (mg/dL)	143 (83–193)
C4 complement (mg/dL)	39 (15–57)
Rheumatoid factor	Negative
Lysozyme (μg/mL)	7.1 (5–11)
Angiotensin-converting enzyme (U/L)	25 (13–100)
IgG (mg/dL)	1434 (694–1618)
IgA (mg/dL)	272 (81–463)
IgM (mg/dL)	184 (48–271)
IgE (kU/L)	447 (≤ 114)
IgG1 (mg/dL)	763 (315–855)
IgG2 (mg/dL)	546 (64–495)
IgG3 (mg/dL)	32 (23–198)
IgG4 (mg/dL)	67.4 (11–157)

No pertinent findings noted

*MPO*: myeloperoxidase; *PR3*: Anti proteinase-3; *ANA*: Antinuclear Antibodies; *p-ANCA*: Perinuclear Anti-neutrophil Cytoplasmic Antibodies; *c-ANCA*: Ancti-neutrophil Cytoplasmic Antibodies; *IgG*: Immunoglobulin G; *IgA*: Immunoglobulin A; *IgM*: Immunoglobulin M; *IgE*: Immunoglobulin E; *IgG1*: Immunoglobulin G1; *IgG2*: Immunoglobulin G2; *IgG3*: Immunoglobulin G3; *IgG4*: Immunoglobulin G4

**Fig. 2:**
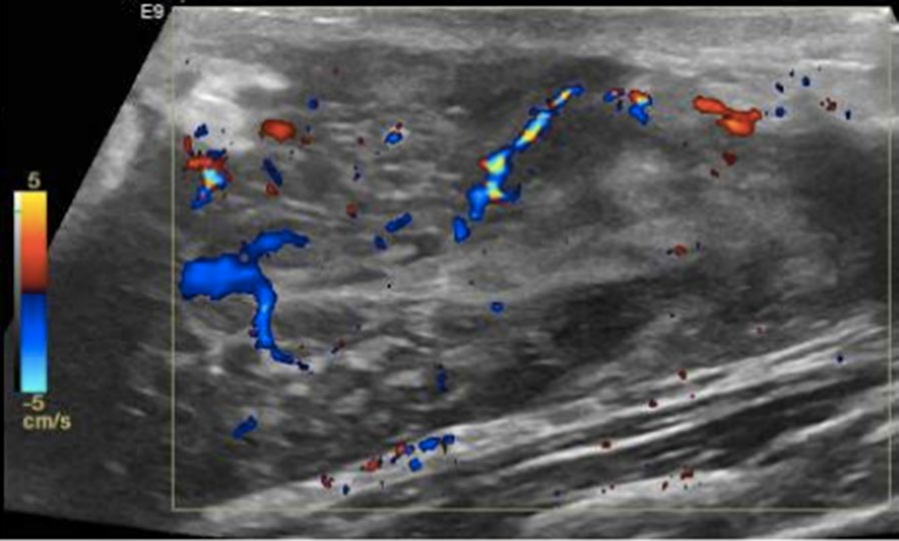
Doppler ultrasound of the patient’s right breast revealed a large, complex area measuring approximately 3.3 cm in thickness with areas of internal vascularity

An ultrasound scan of her right breast revealed a large, complex area measuring approximately 3.3 cm in thickness with areas of internal vascularity (Fig. 2). Upon surgical consultation, needle aspiration of the right breast was performed, and the aspirate fluid was sent for bacterial, fungal, and acid fast (AFB) staining as well as culture. Sonogram guided, vacuum-assisted core biopsy revealed chronic inflammation of the breast with adjacent granulomatous inflammation with neutrophilic microabscess formation. Chest roentgenogram was negative for any mediastinal lymphadenopathy, cardiac, or pulmonary abnormalities. Ophthalmologic examination including slit lamp examination was negative for uveitis and episcleritis. Based on lack of these systemic findings, the diagnosis of idiopathic granulomatous mastitis with erythema nodosum and polyarthritis was made.

During the patient’s hospital course, the skin lesions, on the lower extremities, were noted to progressively improve, with near return to baseline prior to discharge. Culture of the aspirate from breast, was negative for bacterial, fungal, and AFB organisms. Approximately 2 weeks after discharge, the patient was started on a course of prednisone 60 mg daily. Naproxen was stopped after resolution of arthritis and erythema nodosum at that time. With sustained improvement of her symptoms and particularly her breast findings, the patient was placed on steroid taper treatment of approximately 12 weeks duration, which she has been tolerating and responding well to.

## Discussion

This report identified the first adolescent female with a diagnosis of idiopathic granulomatous mastitis (IGM), which is a benign breast disease of unknown etiology. It represents 1.6% of all breast diseases [7] and is considered to be a great imitator as its clinical manifestations mimic many diseases, including sarcoidosis, tuberculosis, malignancy, and infectious organisms. In our case, we performed an extensive workup including assessment of thyroid function, vitamin D levels, antineutrophil cytoplasmic antibodies, double-stranded DNA, rheumatoid factor, antinuclear antibodies, and smooth muscle antibodies with negative/normal results, thereby ruling out autoimmune as well as endocrine causes. Moreover, other possible etiologies such as infectious and rheumatologic conditions were ruled out on the basis of laboratory studies, including complement levels to rule out serum sickness, immunoglobulin (Ig) panel with IgG subclass studies, and interferon gamma release assay (IGRA) to assess for mycobacterial infection, human immunodeficiency virus (HIV), and inflammatory markers. In our patient, all laboratory studies were negative except for elevated ESR and IgE levels (Tables 1 and 3). Absence of pulmonary symptoms, normal ophthalmologic examination, normal serum angiotensin-converting enzyme (ACE), lysozyme, and 25 (OH) vitamin D levels, as well as histopathologic examination substantiated the exclusion of sarcoidosis as a prima facie diagnosis in our patient (Table 2).

Recent publications have suggested possible infectious etiologies in some patients, such as involvement of *Corynebacterium* species resulting in a clinically and histologically similar condition in some patients [8]. Although not reported in humans, exposure to *Mycoplasma bovis* has been attributed to causing an uncannily similar condition in cattle [9]; our patient reported no such exposures, and the Gram stains and cultures from the aspirates were negative. Other published reports suggested carcinomas as part of the differential diagnosis, besides various infectious, autoimmune, and connective tissue diseases [6]. There is, however, stronger evidence supporting the association of IGM with preexisting connective tissue diseases such as Sjögren syndrome [15], rheumatoid arthritis, granulomatosis with polyangiitis, and sarcoidosis [6], none of which was present in our patient.

Our patient’s presentation included extramammary manifestations of arthritis and erythema nodosum. Such association has been described so far in ten cases (Table 4) [10–18]. Interestingly, our patient’s history was negative for all recognized risk factors, including oral contraceptives, breast implants, pregnancy, lactation, and puerperium [9, 11, 12].

**Table 4:** Summary of various case reports found of patients with IGM

Author	Patient age (years)	Risk factor(s)	Race/ethnicity	Treatment
Adams *et al*.	24	Postpartum	Not described	Indomethacin + rifampicin and isoniazid
Binesh *et al*.	40	Parous	Middle Eastern (Iranian)	Dexamethasone followed by prednisolone
Nakamura *et al*.	27	Diabetes mellitus	Asian (Japanese)	Prednisolone
Zabetian *et al*.	43	Hx of BCG vaccination Parous	Hispanic	Prednisone followed by azathioprine
Olfatbakhsh *et al*.	30	Pregnancy (32 weeks)	Middle Eastern (Iranian)	High-dose corticosteroid
Alungal *et al*.	25	None described	Indian	Corticosteroid
Iqbal *et al*.	60	Hx of hormone replacement therapy Rheumatoid arthritis Asthma Pulmonary fibrosis Hiatus hernia ITP	Not described	Conservative management
Salesi *et al*.	23	Pregnant (28 weeks)	Middle Eastern (Iran)	Prednisolone Colchicine Azathioprine
Bes *et al*.	34	None	Middle Eastern (Turkish)	Corticosteroid
Bes *et al*.	27	None described	Middle Eastern (Turkish)	Corticosteroid

Data shown include patient age, risk factors present, race/ethnicity, and treatment modalities that the patients underwent. *BCG Vaccination*: Bacillus Calmetter – Guérin Vaccination; *ITP*: Idiopathic/Immune Thrombocytopenic Purpura; *Hx*: History

In terms of imaging methods to aid in diagnosis, ultrasound may be the best preliminary imaging modality for diagnosis [19], although magnetic resonance imaging (MRI) of breast may help differentiate GM from breast cancers [20]. In our patient, due to younger age and absence of additional findings such as lymphadenopathy and skin changes, the suspicion for malignancy was low. In any case, the gold standard for diagnosis is histopathologic confirmation.

Many treatment modalities have been described but have had varying degrees of success. These options include watchful waiting, surgery, systemic corticosteroids, and chemotherapeutic agents [2–7, 10–18]. Glucocorticoid treatment is the mainstay of therapy as it has been proven to cause significant disease regression [21]. Furthermore, its use has allowed for a more conservative approach, leading to decreased morbidity and favorable outcomes [2]. Surgical treatment is to be avoided as much as possible in nonrecurrent disease owing to auspicious results using conservative measures and histologically benign nature of the illness. In our case, the extraarticular manifestations of erythema nodosum and arthritis showed significant improvement with naproxen within 2 weeks and her breast swelling improved significantly within 1–2 weeks of corticosteroid treatment.

Pandey *et al*. [2] studied the use of corticosteroid therapy and followed treatment outcomes in the largest North American cohort of patients with IGM (*n* = 49). Ninety percent of their cohort was prescribed systemic corticosteroids, with the other members of their cohort either receiving surgical management or being observed for spontaneous resolution. Of those that received steroids, 80% were found to have complete resolution of disease. In this cohort, median time to complete resolution was 159 days (interquartile range 120–241 days).

Chemotherapeutic agents, such as azathioprine and methotrexate (MTX), are of benefit for several reasons, including facilitating tapering of steroids, in cases refractory to steroid therapy and in cases of recurrence [22]. Kim *et al*. reported favorable outcome in using MTX in five women with granulomatous mastitis [23]. A review of 112 cases by Akbulut *et al*. [22] demonstrated that MTX was useful as it prevented complications, resolved the inflammatory process, and limited side effects associated with prolonged, high-dose corticosteroid use.

## Conclusion

Our patient represents the first documented case of IGM in the pediatric age group. This case is particularly impactful owing to the presence of extramammary manifestations such as erythema nodosum and arthritis as well as the absence of known risk factors. It is paramount to exclude the treatable, secondary causes of GM as outlined above. As evident in our case, as well as literature on adult patients, patients do well with a tapered corticosteroid therapy over several weeks. Due to the rarity and heterogeneity of this condition, we postulate that it is prudent to keep an “open mind” to the disease evolving to another granulomatous entity, such as sarcoidosis or Crohn’s disease.

## Data Availability

Data sharing is not applicable to this article as no datasets were generated or analyzed during the current study.

## Abbreviations

GM: Granulomatous mastitis
MTX: Methotrexate
Vit D 25 (OH): 25-Hydroxy vitamin D
